# Complete Genome Sequence of Lactobacillus reuteri Byun-re-01, Isolated from Mouse Small Intestine

**DOI:** 10.1128/MRA.00984-18

**Published:** 2018-11-01

**Authors:** Dongjun Kim, Mun-ju Cho, Seungchan Cho, Yongjun Lee, Sung June Byun, Sukchan Lee

**Affiliations:** a Department of Genetic Engineering, Sungkyunkwan University, Jangan-gu, Suwon, Republic of Korea; b Animal Biotechnology Division, National Institute of Animal Science, Rural Development Administration, Kongjwipatjwi-ro, Iseo-myeon, Wanju-Gun, Jeollabuk-do, Republic of Korea; University of Delaware

## Abstract

Lactic acid bacteria (LAB) are generally recognized as safe (GRAS) and serve as probiotic bacteria when consumed in adequate amounts. Here, we report the complete genome sequence of Lactobacillus reuteri Byun-re-01, isolated from mouse small intestine.

## ANNOUNCEMENT

Lactobacillus spp. generally inhabit the gastrointestinal (GI) and urogenital tracts, conferring beneficial effects to the host by inhibiting the proliferation of pathogens and modulating the immune system (1). Expression of recombinant proteins in a *Lactobacillus* sp. could increase its probiotic activity. As revealed by our previous research, Lactobacillus casei ATCC 334 expressing 3D8 scFv (a nucleic acid-hydrolyzing antibody) exerted an antiviral effect on murine norovirus (2). It also showed enhanced probiotic activity in the intestines of mice by increasing the population of Pediococcus acidilactici, another probiotic (3). Previous studies have thus confirmed that lactic acid bacteria expressing 3D8 scFv could serve as an antiviral drug. Our ultimate goal is to develop intestinal *Lactobacillus* spp. expressing 3D8 scFv as an antiviral food additive. Most lactic acid bacteria survive in the gastrointestinal (GI) tract under anaerobic conditions; thus, they are already commonly used as probiotics (4).

Here, we present the complete genome of Lactobacillus reuteri Byun-re-01, isolated from the duodenum of a 6-week-old female specific-pathogen-free ICR mouse (DBL, Republic of Korea). All animal procedures performed in this study were reviewed, approved, and supervised by the Institutional Animal Care and Use Committee (IACUC) of Konkuk University (KU16080). All experimental procedures were in accordance with the guidelines of the Institute of Laboratory Animal Resources (ILAR).

Tissue from the duodenum was isolated and homogenized using 1.6-mm stainless steel beads. Lactic acid bacterial isolates were cultured in de Man-Rogosa-Sharpe (MRS) medium and selected based on their rod-shaped morphology under a light microscope. Comparisons of 16S rRNA and DNA gyrase subunit B of these isolates revealed 99% similarity to those of most other L. reuteri strains.

Genomic DNA was extracted by treating cells with lysozyme and mutanolysin, followed by use of a G-spin genomic DNA extraction kit (iNtRON, Republic of Korea) (5). The L. reuteri Byun-re-01 genome was sequenced at Macrogen (Seoul, Republic of Korea) using both the Illumina HiSeq 2000 (2 × 100-bp paired-end sequencing) and PacBio RS II (Pacific Biosciences, USA) platforms. Library preparation for Illumina and PacBio sequencing was performed using the NEBNext Ultra DNA library prep kit for Illumina (NE, USA) and the PacBio DNA template prep kit 1.0 (Pacific Biosciences, USA), respectively, according to the manufacturers’ instructions. The library insert sizes were 325 bp for Illumina sequencing and 20 kb for PacBio RS single-molecule real-time (SMRT) sequencing. Ultimately, 68,530,004 paired-end reads were generated by Illumina sequencing, and 128,652 long reads were generated by PacBio sequencing. Trimmed reads generated by Trimmomatic 0.32 software (6) were used for *de novo* assembly based on the hierarchical genome assembly process (HGAP3) using SMRT Analysis software version 2.3.0 (7). To obtain a high-quality sequence, error correction of the assembled contig was performed by hybrid assembly using Illumina raw sequence data. This resulted in one contig representing a complete chromosome sequence (*N*_50_, 2,244,514 bp; final coverage, 404×). The complete genome of L. reuteri Byun-re-01 is 2,244,514 bp, and its G+C content is 38.9%. The genome was annotated using Prokka software version 1.12b, which continuously performs a series of annotation processes (8). The genome of L. reuteri Byun-re-01 contains 72 tRNA genes and 18 rRNA genes. A total of 2,083 protein-coding sequences were discovered.

### Data availability.

The chromosomal sequence of Lactobacillus reuteri Byun-re-01 has been deposited in GenBank under the accession number CP029613.
